# Severe Stunting Symptoms upon Nepovirus Infection Are Reminiscent of a Chronic Hypersensitive-like Response in a Perennial Woody Fruit Crop

**DOI:** 10.3390/v13112138

**Published:** 2021-10-22

**Authors:** Isabelle R. Martin, Emmanuelle Vigne, Amandine Velt, Jean-Michel Hily, Shahinez Garcia, Raymonde Baltenweck, Véronique Komar, Camille Rustenholz, Philippe Hugueney, Olivier Lemaire, Corinne Schmitt-Keichinger

**Affiliations:** Santé de la Vigne et Qualité du Vin, INRAE, Université de Strasbourg, 68000 Colmar, France; emmanuelle.vigne@inrae.fr (E.V.); amandine.velt@inrae.fr (A.V.); jean-michel.hily@vignevin.com (J.-M.H.); shahinez.garcia@inrae.fr (S.G.); raymonde.baltenweck@inrae.fr (R.B.); veronique.komar@inrae.fr (V.K.); crustenholz@unistra.fr (C.R.); philippe.hugueney@inrae.fr (P.H.); olivier.lemaire@inrae.fr (O.L.)

**Keywords:** contrasting phenotypes, grapevine, hypersensitive response, metabolome, pathogenicity, plant virus, family *Secoviridae*, transcriptome, virus resistance

## Abstract

Virus infection of plants can result in various degrees of detrimental impacts and disparate symptom types and severities. Although great strides have been made in our understanding of the virus–host interactions in herbaceous model plants, the mechanisms underlying symptom development are poorly understood in perennial fruit crops. Grapevine fanleaf virus (GFLV) causes variable symptoms in most vineyards worldwide. To better understand GFLV-grapevine interactions in relation to symptom development, field and greenhouse trials were conducted with a grapevine genotype that exhibits distinct symptoms in response to a severe and a mild strain of GFLV. After validation of the infection status of the experimental vines by high-throughput sequencing, the transcriptomic and metabolomic profiles in plants infected with the two viral strains were tested and compared by RNA-Seq and LC-MS, respectively, in the differentiating grapevine genotype. In vines infected with the severe GFLV strain, 1023 genes, among which some are implicated in the regulation of the hypersensitive-type response, were specifically deregulated, and a higher accumulation of resveratrol and phytohormones was observed. Interestingly, some experimental vines restricted the virus to the rootstock and remained symptomless. Our results suggest that GFLV induces a strain- and cultivar-specific defense reaction similar to a hypersensitive reaction. This type of defense leads to a severe stunting phenotype in some grapevines, whereas others are resistant. This work is the first evidence of a hypersensitive-like reaction in grapevine during virus infection.

## 1. Introduction

The development of severe symptoms, rather than the presence of the virus itself, constitutes the main nuisance caused by viral infections in crop plants. It is now generally accepted that virus–host interactions are responsible for the development of symptoms, rather than the competition for resources [1,2,3,4]. These specific interactions between the viral pathogenicity determinants and the host components are thought to perturbate the host physiology [3]. The mechanisms underlying pathogenesis are diverse and include RNA silencing when the RNAs from the virus sequences share similarities with endogenous, coding or noncoding, RNAs [2,5,6,7,8,9]. However, the symptoms are mainly determined by viral pathogenicity proteins through protein–protein interactions with a host factor [10,11]. While many viral pathogenicity factors, and their mutants, affecting symptom severity have been identified [12,13,14,15,16,17,18,19,20,21,22], examples of the interactant host factors are rare and, more importantly, the underlying mechanisms have not been described [10,23,24,25]. Moreover, virus–host studies have essentially been conducted on model plants of the genus *Arabidopsis* [23] or *Nicotiana* [10,24,25].

Grapevine is a perennial woody fruit crop of high value in many countries. It hosts more than 80 viruses [26], of which grapevine fanleaf virus (GFLV, genus *Nepovirus*, family *Secoviridae*) is the most detrimental worldwide [27]. The symptoms induced by GFLV vary according to the viral strain, vine genotype, and environmental conditions [28]. They range from foliar discolorations to severe deformations and stunting [28,29,30]. So far, the viral determinants of the symptomatology remain elusive and GFLV-grapevine interactions for pathogenicity are poorly understood. GFLV is a nonenveloped isometric soil-borne virus that is specifically transmitted from vine to vine by the ectoparasitic nematode, *Xiphinema index* (for a review, see [29,31]). Its genome is composed of two single-stranded positive-sense RNAs, both being required for systemic infection. Each RNA encodes a polyprotein that is cleaved by the viral proteinase into functional mature proteins. RNA1 gives rise to five, potentially six, proteins involved in replication and polyprotein processing: protein 1A possibly further cleaved into the X1 and X2 of an unknown function; the helicase-motif containing-1B^Hel^; the small genome-linked protein 1C^VPg^; the proteinase 1D^Pro^; and the RNA-dependent RNA polymerase, 1E^Pol^. RNA2 produces three proteins: the homing protein 2A^HP^ involved in RNA2 replication; the tubule-forming movement protein, 2B^MP^; and the structural protein, 2C^CP^ (for a review, see [29,31]).

In addition to infecting the *Vitis* species, GFLV also infects herbaceous plants, either under vineyard conditions [32,33], or in experimental settings, following mechanical inoculation. Using recombinant constructs between GFLV strains expressing different phenotypes in the *Nicotiana* species, we previously identified the polymerase 1E of strain GHu as a symptom determinant in the compatible hosts, *N. benthamiana* and *N. clevelandii* [14,34]. We also showed that a hypersensitive reaction (HR) is responsible for necrotic symptoms induced by GFLV strain F13 in *N. occidentalis* and identified protein 2A as the avirulence factor. This reaction leads to variable phenotypes in the uninoculated apical leaves, ranging from asymptomatic to necrotic and distorted blades [35].

Mechanistic studies on the symptom development in grapevine are complicated because of, (i) the prevalence of multiple virus infections that makes it difficult to attribute a phenotype to a given virus genotype, and (ii) the lack of an easy and reliable inoculation technique that hinders reverse genetic experiments [36]. Here, we describe a comparative study of the *Vitis vinifera* cultivar (cv.) Gewurztraminer exhibiting differential symptoms upon infection by two different GFLV strains in controlled field and greenhouse conditions. The transcriptomic and metabolomic profiles reveal commonly and differentially regulated *Vitis* genes in response to a mild or a severe GFLV strain and suggest a hypersensitive-like reaction associated with the severe strain.

## 2. Materials and Methods

### 2.1. Biological Material and Virus Inoculation

The GFLV strains, F13, GHu, B844, CO1, and CO2 (Supplemental Table S1), from different *Vitis vinifera* cvs [34,35,37,38,39], were used in this study. All strains are composed of one RNA1 and one RNA2, except strain B844, which contains two RNA2 molecules designated RNA2a and RNA2b (Table S1). After multiple passages on herbaceous hosts, these five GFLV strains were transferred to the certified rootstock Kober 5BB clone 259 (*Vitis berlandieri* × *Vitis riparia*) by in vitro heterologous grafting. Briefly, rootstock cuttings were sterilized and assembled in tissue culture onto the stem fragments of *Chenopodium quinoa* infected by a specific GFLV strain, following mechanical inoculation, to allow the virus to translocate from the herbaceous host into the grapevine rootstock [37]. After removal of the *C. quinoa* stems, the grapevine rootstocks were further grown and micropropagated in tissue culture. The rootstocks were then tested for GFLV infection and the infected rootstocks were grafted with *V. vinifera* cv. Gewurztraminer (Gw; certified clone 643), or Chardonnay (Ch; certified clone 131), using green-grafting in an environmental growth chamber [40]. Each test vine consisted of a scion cultivar (Gw or Ch) grafted onto the Kober 5BB rootstock and will be named, herein, after the scion cultivar (Gw or Ch). The plants of Gw and Ch corresponding to scions grafted onto uninoculated rootstocks were used as controls. A total of 131 vines were established in four trials, as detailed in Table S2, either in an *X. index*-free field site or in a greenhouse. The vines in the field were trained according to the simple Guyot method. In the greenhouse, the plants were in individual pots and trained with two shoots at a 1.80 m height.

### 2.2. Symptom Scoring

Leaf discoloration including yellowing, variegation, mottling, mosaic, and vein banding was visually estimated in June and scored from 0 to 4 (0: no discoloration; 1 to 4: 1–25%, 25–50%, 50–75%, and 75–100% of the leaves showing discoloration, respectively). Leaf deformation corresponding to a fan-like aspect, asymmetric blades, or small leaves was scored in June from 0 to 4 (0: no deformation; 1 to 4: 1–25%, 25–50%, 50–75%, and 75–100% of the leaves showing deformation, respectively). Development abnormalities, including double nodes, abnormal bifurcations, fasciation, and zigzagging of the shoots were estimated in June and scored from 0 to 4 (0: no abnormality; 1 to 4: 1–25%, 25–50%, 50–75%, and 75–100% of the leaves showing abnormalities). Stunting was evaluated in June from 0 to 4 (0: no stunting; 1: low; 2: medium; 3: strong; and 4: very strong). The control plant development served as a reference (no stunting) and the training wires were used to estimate the height of the plants. Coulure (flower abortion) was scored in July from 0 to 3 (0: no coulure; 1: low; 2: medium; and 3: strong). A medium score corresponded to flower abortion observed in half of the clusters, while a strong score was similar to the reference, Muscat Ottonel, of our collection, a cultivar known for its sensitivity to flower abortion.

### 2.3. Detection of GFLV

The translocation of the virus from the rootstock to the cultivar was monitored over time by a double antibody sandwich-enzyme-linked immune-sorbent assay (DAS-ELISA) of leaf samples using a GFLV-specific serum (Bioreba, Reinach, Switzerland). For an estimation of the virus titer, the absorbance values were compared with the known amounts of purified GFLV serially diluted in the sap of healthy grapevines [34,35]. The presence of GFLV RNAs was confirmed by an immunocapture-reverse transcription-polymerase chain reaction-restriction fragment length polymorphism assay (IC-RT-PCR-RFLP), using *Sty*I as described in [41]. The detection of viral siRNAs by northern blot was performed on RNA isolated from cortical scrapings and leaves as described in [42]. Long viral RNAs were detected by RT-qPCR [42].

### 2.4. RNA Extraction and RNA-Sequencing (RNA-Seq)

The total RNA from the leaf samples (200 mg) was isolated using the RNeasy Plant Mini Kit (Qiagen, Hilden, Germany), according to the manufacturer’s instructions, with the addition of DNase. All library preparations, RNA treatments, and RNA sequencing steps were performed by the GeT-PlaGe platform of Genotoul (Genopole, Toulouse, France) using the TruSeq Stranded mRNA sample prep kit (Illumina, San Diego, CA, USA). Using poly(A) selection, the mRNAs were isolated, fragmented, and reverse-transcribed. Experiments were performed on an Illumina HiSeq 3000 (Illumina, San Diego, CA, USA), using a paired-end read length of 2 × 150 base pairs with Illumina Hiseq3000/4000 SBS sequencing kits.

### 2.5. Sample Infection Status and GFLV Diversity

Analyses of the sequence datasets were performed using CLC Genomics Workbench 11.0 software (Qiagen Digital Insights, Aarhus, Denmark), as described in [43]. After the trimming and quality check, only reads longer than 70 nucleotides were kept. Using loose parameters, with a length fraction of 0.5 and similarity of 0.7, a direct mapping was performed with a curated set of references based on grapevine viruses and viroids [27]. To study the genetic diversity, the de novo assembly tool from CLC Workbench (Qiagen Digital Insights, Aarhus, Denmark) was used and the contigs were mapped against GFLV-coding regions. The GFLV RNA1 and RNA2 5′ and 3′ untranslated regions (UTRs) were excluded in this study because they contain sequences shared by the two RNAs [29,44]. Multiple rounds of mapping and assembly were implemented until complete coding regions were obtained.

Alignment analysis of the nucleic acid sequences and neighbor-joining-based phylogenetic trees were performed using CLC Workbench, with bootstrapping analyses of 1000 replicates.

### 2.6. Differential Gene Expression (DEG) Analysis

Raw reads were aligned on the *Vitis vinifera* PN40024 (12X.v2) reference genome [45,46], using TopHat2 (v.2.0.11) [47] and Bowtie2 (v.2.2.1) [48], with the exception of the CRIBI V2.1 gene annotation, which was preferred to VCost.v3 because its use is more widespread. The gene expression quantification was performed with HTSeq-count (v.6.0.0) [49]. The count normalization and detection of differentially expressed genes was done using R (v.3.3.2) and the DESeq2 package. The differential gene expression of the replicates for each condition (GFLV-B844 or GFLV-F13-inoculated) compared to the control replicates, were analyzed. The *p*-values were adjusted with the Benjamini–Hochberg procedure [50]. The genes were considered differentially expressed when the false discovery rate was below 5% (FDR ≤ 0.05).

Gene categorization was performed by an enrichment of the Gene Ontology (GO) terms on the DEGs using the TopGo R package (v2.32.0) [51]. Revigo online-specific tools were used to hierarchize the enriched GOs, providing a more suitable representation [52].

### 2.7. Reverse Transcription Quantitative PCR (RT-qPCR)

Specific primers (Table S3) were designed from sequences retrieved from the RNA-Seq data. The reverse transcription step was performed on 1 µg of total RNA using Superscript III enzyme (Invitrogen, Carlsbad, CA, USA) and oligo-d(T) primer. Real-time quantitative PCRs were carried out using a LightCycler480 thermocycler and a LightCycler 480 SYBR Green I Master (both from Roche, Basel, Switzerland). The thermal cycling conditions were: 5 min at 95 °C; 55 cycles of 10 s at 95 °C; 15 s at 60 °C; 15 s at 72 °C; and a final step of 30 s at 40 °C. Primer specificity was checked by a melting curve at the end of each cycle. The results were normalized to the expression of the more stable gene defined with geNorm [53] and Norm Finder [54] software, namely, *NEMFI*. The primer efficiency was determined by LinRegPCR software (v.2017.0) [55]. We used the Pfaffl method [56] to calculate the relative expression, using the control samples as calibrators.

### 2.8. Metabolomic Analyses

Ultrahigh-pressure liquid chromatography and mass spectrometry (UHPLC-MS) analyses were performed as described previously [57], with some modifications. Metabolites were extracted with methanol (25 µL/mg frozen fresh leaves) supplemented with an external standard (apigenin, 1 mg/L). An amount of 50 mg of each sample were sonicated in an ultrasonic bath (Fisher Scientific, Hampton, NH, USA) for 15 min. After centrifugation at 12,000× *g* for 10 min, 200 µL of the supernatants were recovered for LC-MS analyses. The analyses were performed using a Dionex Ultimate 3000 UHPLC system (Thermo Fisher Scientific, Waltham, MA, USA). The chromatographic separations were performed on a Nucleodur C18 HTec column (150 × 2 mm, 1.8 μm particle size; Macherey-Nagel, Düren, Germany) maintained at 30 °C. The mobile phase consisted of acetonitrile/formic acid (0.1%, *v*/*v*) (eluant A), and water/formic acid (0.1%, *v*/*v*) (eluant B), at a flow rate of 0.25 mL/min. The gradient elution program was as follows: 0 to 4 min, 80% to 70% B; 4 to 5 min, 70% to 50% B; 5 to 6.5 min, 50% B isocratic; 6.5 to 8.5 min, 0% B; and 8.5 to 10 min, 0% B isocratic. The sample volume injected was 1 μL. The UHPLC system was coupled to an Exactive Orbitrap mass spectrometer (Thermo Fischer Scientific, Waltham, MA, USA), equipped with an electrospray ionization (ESI) source operating in positive or negative mode. The parameters were set at 360 °C for the ion transfer capillary temperature, and 3500 V (positive mode) and 2500 V (negative mode) for the needle voltages. Nebulization with nitrogen sheath gas and auxiliary gas were maintained at 60 and 15 arbitrary units, respectively. The spectra were acquired within the *m/z* mass range of 110 to 1200 atomic mass units (a.m.u.), using a resolution of 50,000 at *m/z* 200 a.m.u. The system was calibrated internally using dibutyl phthalate as lock mass at *m/z* 279.1591, giving a mass accuracy lower than 1 ppm in the positive mode. The system was calibrated externally using calibration solution (Pierce™ LTQ ESI Negative Ion solution, Thermo Fisher Scientific), giving a mass accuracy lower than 5 ppm in the negative mode. The instruments were controlled using the Xcalibur software. The exact *m/z* and retention time of each metabolite were used for targeted metabolomic analyses using the Xcalibur software. A targeted metabolomic strategy was used to quantify the relative amounts of a total of 101 metabolites in grapevine leave extracts. The selected metabolites were grouped into eight major chemical or functional families, including amino acids and derivatives, flavonoids, hormones, hydroxycinnamic acids and derivatives, organic acids, proanthocyanidins, stilbenoids, and terpenoids. For some metabolites, identity was confirmed with the corresponding authentic standard provided by Sigma-Aldrich (Saint-Quentin-Fallavier, France) or Extrasynthese (Genay, France).

## 3. Results

### 3.1. Differential Symptoms in Vitis vinifera cv. Gewurztraminer Infected by Five Different GFLV Strains

**Figure 1:**
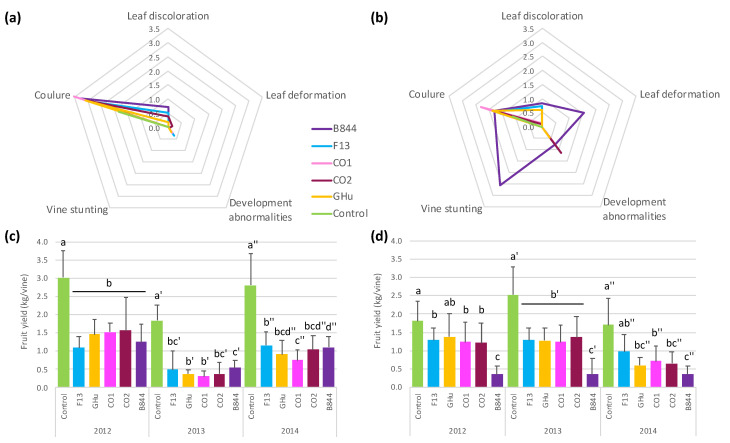
Analyses of symptoms (**a**,**b**) and fruit yield (**c**,**d**) on *Vitis vinifera* ‘Chardonnay’ (Ch) (**a**,**c**) and ‘Gewurztraminer’ (Gw) (**b**,**d**) vines infected with five different strains of grapevine fanleaf virus (GFLV). The five strains caused similar symptoms and fruit yield reduction on Ch vines, whereas strain B844 was more severe than the strains F13, CO_1_, CO_2_ and the GHu on Gw vines. Six to eight plants of each vine-virus combination were monitored over three growing seasons and the results are expressed as mean values. (**a**,**b**) Spider graphs represent the visual scoring of general symptoms from 2012 to 2014. Leaf deformation corresponds to the fan-like appearance and small size of the limb. Development abnormalities of the shoots include double nodes, abnormal bifurcations, and fasciation. Coulure qualifies flower abortion. (**c**,**d**) Histograms represent the average fruit production per plant. Different letters indicate significant differences (Mann–Whitney test, *p* < 0.05). Different signs (no sign, ’ and ”) are for different years.

**Figure 2:**
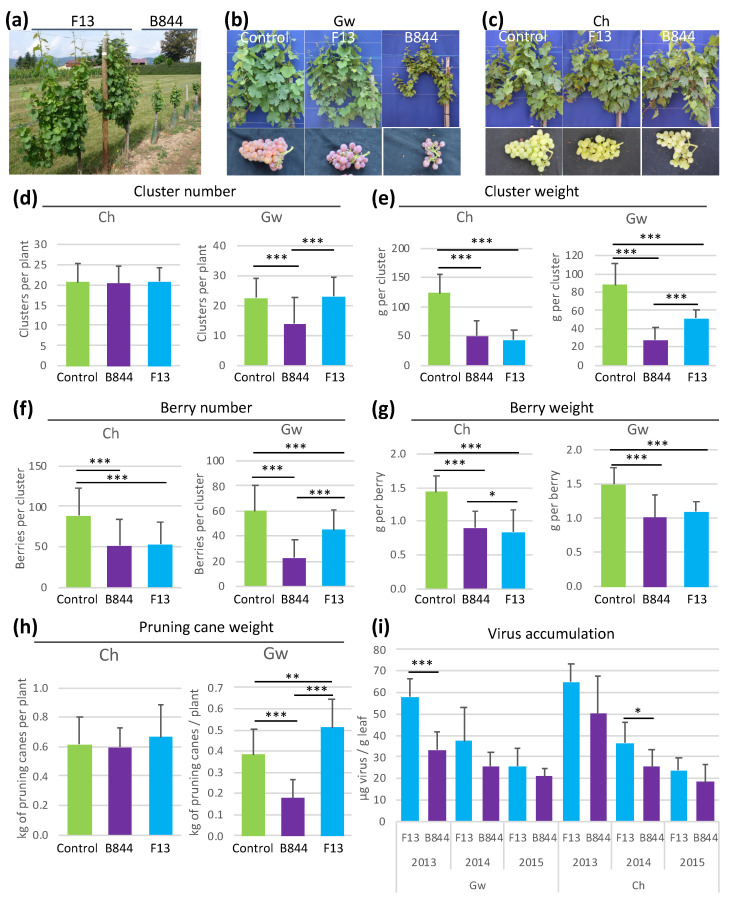
Grapevine fanleaf virus (GFLV) strains B844 and F13 induce contrasting phenotypes on *Vitis vinifera* cv. Gewurztraminer (Gw) vines. (**a**) plants were photographed in spring 2011. (**b**) Gw plants and fruits photographed in June and September 2012, respectively, show the drastic effect of strain B844. (**c**) ‘Chardonnay’ (Ch) plants and fruits photographed in June and September 2012, respectively, show similar effects of the two GFLV strains. (**d**–**h**) Ch and Gw vines infected with GFLV strains F13 and B844 were monitored for cluster number (**d**), cluster weight (**e**), berry number (**f**), berry weight (**g**), and pruning wood weight (**h**), from 2012 to 2014. Virus accumulation (**i**) was estimated by semiquantitative DAS-ELISA from 2013 to 2015. Error bars = standard deviation. Statistically significant differences (nonparametric Mann–Whitney test) are indicated with asterisks (* *p* < 0.05, ** *p* < 0.01, *** *p* < 0.005).

To gain insights into the GFLV symptom determinants, the vines infected with a single GFLV strain were produced using a two-step grafting procedure to separately transfer five GFLV strains (F13, GHu, B844, CO_1_ and CO_2_, Table S1) from mechanically inoculated *C. quinoa* plants into scions of cultivar Chardonnay (Ch) or Gewurztraminer (Gw) grafted onto rootstock Kober 5BB. Non-inoculated graft assemblies served as controls. In the first trial (Trial 1, Table S2), six to eight vines of each cultivar-virus strain combination (for a total of 90 vines) were established in an experimental vineyard devoid of the nematode vector, *X. index,* that was maintained according to local commercial practices. From 2012 to 2014, the symptoms were visually scored and the fruits were collected at harvest time. All of the GFLV strains induced symptoms in both the Ch and Gw vines. On the Ch vines, all strains essentially caused flower abortion, known as coulure, leaf discolorations, such as mosaic and mottling, and a reduction in the fruit yield (Figure 1, Table S4). The yield reduction was more variable over time than among strains (Figure 1c, Table S5). On Gw vines, all strains induced coulure and very similar discolorations; however, strain B844 induced leaf deformation and a severe stunting (Figure 1b). These symptoms were already visible during the third growing season (in 2009) and persisted over time (Figure 2a,b). Moreover, strain B844 had a more severe effect on the fruit yield compared with the other strains, with the reduction consistently reaching approximately 80% (Figure 1d, Table S5). Since strain F13 behaved similarly to strains GHu, CO1, and CO_2_ on both grapevine cultivars, subsequent work focused on GFLV-F13 and GFLV-B844.

Starting in 2012, we quantified the phenotypes of vines infected by GFLV-F13 or GFLV-B844, in addition to visually scoring the symptoms (Figure 1). Six to eight plants per plant-virus combination were monitored for at least three years. The results are presented as cumulative data in Figure 2 (and in detail, according to years, in Figure S1). On the Ch vines, both strains affected fruit production by reducing the mean cluster weight, the mean number of berries per cluster, and the mean weight of the berries, but not the mean number of clusters (Figure 2d–g). No significant difference was consistently observed between the two strains. On the contrary, strains F13 and B844 caused distinct phenotypes on the Gw vines. Strain F13 had fairly the same effect on Ch vines, whereas B844 caused a more pronounced reduction of the mean cluster weight and number of berries per cluster. It also occasionally caused a reduction of the mean number of clusters per plant (Figure 2d–g). The measurements of pruning wood weight in winter, as an indication of vine growth during the preceding season [58,59], revealed a differential effect of the two strains on Gw vines: while GFLV-B844 induced a 50% reduction compared with uninfected vines, GFLV-F13 increased the wood weight by a factor of 1.2 to 1.3 (Figure 2h, Table S6). In summary, strain B844 had a more drastic effect than strain F13 on fruit production in Gw vines and drastically reduced growth.

To test whether the symptom severity was related to the virus titer, the level of virus accumulation was measured by semiquantitative DAS-ELISA in the young leaves of Ch and Gw scions (Figure 2i). Over the years, the virus titer was quite variable, but generally not significantly different, between the two strains (although GFLV-B844 always accumulated less than GFLV-F13), suggesting that virus accumulation is not directly associated with symptom severity. The control vines always tested negative for GFLV. Altogether, this work allowed us to identify B844 and F13 as severe and mild strains of GFLV on Gw grapevines.

### 3.2. RNA-Sequencing and Read Mapping on Virus and Grapevine Genomes

To shed light on the processes underlying the onset of mild or severe symptoms, the transcriptomes of F13- and B844-infected Gw grapevines were compared to those of the uninoculated controls. We sampled leaves after budburst, in the spring of 2016, on similar shoots and at a similar phenological stage (visible clusters, 4 to 6 expanded leaves). Three plants were analyzed per treatment: non-inoculated Gw scions (control, vines n° B31, B34, and C51), GFLV-F13 infected Gw scions (F13, vines n° B52, B53, and C38), and GFLV-B844-infected Gw scions (B844, vines n° B47, C39, and C40). Representative pictures of the three treatments are shown in Figure S2A. To characterize the stunting symptoms, we counted the open leaves and measured the size of the leaves and shoots. The results (Figure S2b) show that plants B47 and C40 infected by GFLV-B844 developed fewer leaves, and that these leaves, and their corresponding shoots, were shorter compared to non-inoculated plants. It is worth noting that the plants inoculated with strain B844 exhibited more variable phenotypes than the plants of the other two treatments.

The total RNA extracted from the leaves was subjected to sequencing. For each plant, 52 to 85 million paired-end reads of 150 nucleotides were obtained, with the exception of plant B47 (B844-inoculated), for which we obtained 165 million reads, indicating an overamplification (Table 1 and Table S7 and Table S8).

#### 3.2.1. Infection Status of the Plant Samples

**Figure 3:**
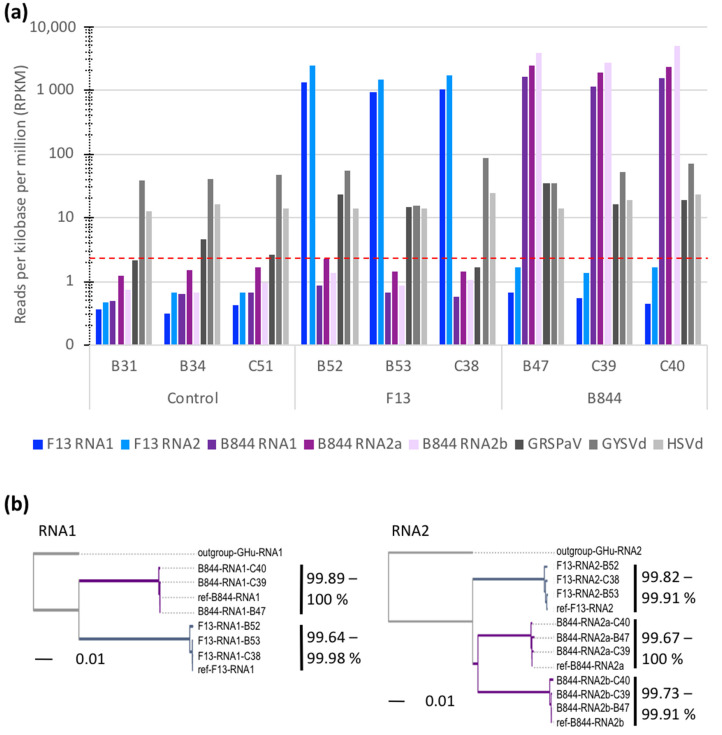
Analysis of virus and viroid sequences retrieved from uninoculated (control), and grapevine fanleaf virus (GFLV) strain F13 (F13)- or B844 (B844)-inoculated *Vitis vinifera* cv. Gewurztraminer (Gw) scions. (**a**) Distribution of reads matching grapevine virus or viroids in three plants for each treatment. The number of reads was normalized to the depth of sequencing and the length of the viral/viroidal genomes. The dotted red line indicates the intra-lane contamination threshold. (**b**) Phylogenetic relationships of GFLV genomes assembled from sequences obtained from leaf samples of infected scions. Alignments based on the maximum likelihood of the coding sequence of GFLV RNA1 and 2 (excluding the 5′ and 3′ UTRs) were performed using CLC Workbench. Strain GHu of GFLV serves as an outgroup. Ref-F13 and ref-B844 represent sequences available in GenBank (see Table S1 for accession numbers).

To establish the virome of the vines, the trimmed reads were aligned to known virus and virus-like sequences found in grapevine. The vast majority of sequences matched to GFLV (Figure 3 and Table S7). Intra-lane contamination, with a low quantity of non-expected GFLV sequences, as previously reported in [42,43], was found in all samples. In addition, grapevine rupestris stem-pitting-associated virus (GRSPaV), grapevine yellow speckle viroid (GYSVd), and hop stunt viroid (HSVd) were detected. This virus and two viroids are ubiquitous in the grapevine worldwide [60].

To further confirm the identity of GFLV sequences, the reads matching the GFLV genome were de novo assembled. The resulting contigs, corresponding to viral genomic sequences, were named after the identification of the plant they originated from. Phylogenetic trees were then built by aligning RNA1 and RNA2 coding sequences. The sequences retrieved from the plants inoculated with GFLV-F13 (B52, B53, and C38), or GFLV-B844 (B47, C39, and C40), were grouped together and shared more than 99.64% of their nucleotide sequence identity with the GFLV-F13 and GFLV-B844 reference sequences, respectively (Figure 3b). Thus, the reads mapping to the GFLV sequences indicated a perfect correlation with the inoculated GFLV strain, and a genome diversity within the range observed earlier that is attributable to the quasi-species nature of the viruses [41,43].

Altogether, these results show that the analyzed samples were confidently infected with the expected GFLV strains, and no superinfection took place during the course of the experiment.

#### 3.2.2. Grapevine Transcriptome Analysis

- Mapping to the grapevine reference genome.

The sequence reads were aligned against the grapevine reference genome [61,62]. A total of 82 to 88% reads mapped to the grapevine genome (Table 1 and Table S8) and 81 to 86% corresponded to uniquely mapped reads. A total of 77 to 82% reads were assigned to a PN40024 gene. Globally, reads were obtained for 25,255 genes, representing about 80% of the total (31,842) annotated genes of the reference genome.

**Figure 4:**
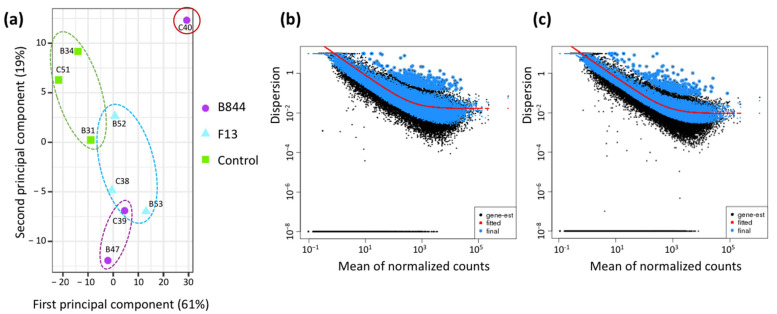
Global analysis of RNA-Seq analyses of uninoculated (control), and grapevine fanleaf virus (GFLV) strain F13 (F13)- or B844 (B844)-inoculated *Vitis vinifera* cv. Gewurztraminer. (**a**) Principal component analysis of total expressed genes after variance-stabilized transformation showing the good grouping of eight samples with two components explaining 80% of the variability and the erratic behavior of sample C40 (GFLV-B844-infected Gw scion). (**b**,**c**) Dispersion estimates of reads before (**b**) and after (**c**) removal of sample C40.

With the exception of plant C40 (GFLV-B844-infected), the samples were grouped according to the experimental conditions, as seen by the principal component analysis (PCA) based on the read counts after variance-stabilized transformation (VST) (Figure 4a). Variability was observed between the biological replicates of a given treatment. However, the treatments were more discriminant than the replicates. It is worth noting that the differences were more prominent between the control and infected vines than between vines inoculated by the two GFLV strains. Because sample C40 was an outlier, we decided to exclude it from further analyses. The estimation of dispersion showed that no information was lost by eliminating this sample, and a narrower estimated dispersion resulted (Figure 4b,c).

- Gw genes differentially expressed during GFLV-F13 and GFLV-B844 infections.

**Figure 5:**
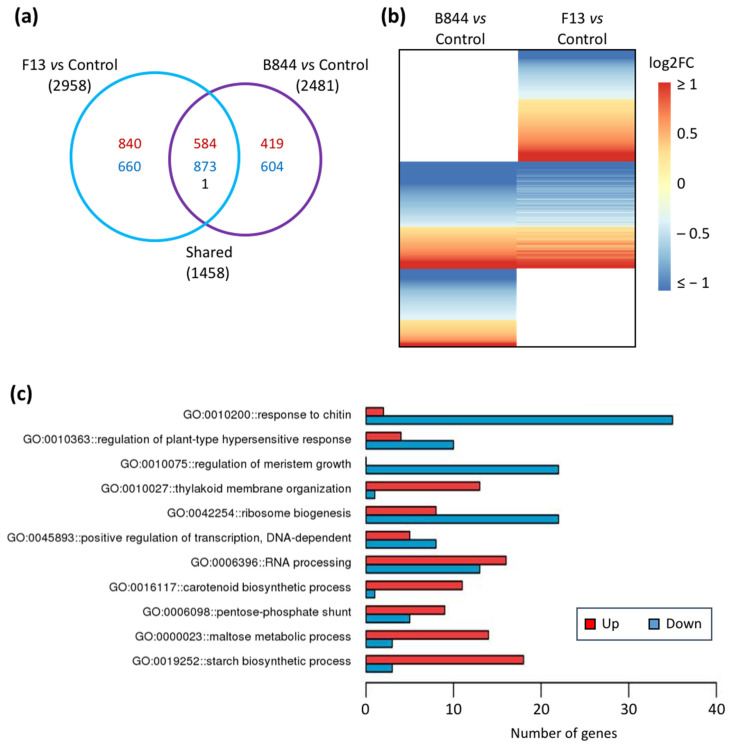
Global analysis of transcriptomic changes upon grapevine fanleaf virus (GFLV) infection. (**a**) Venn diagram displaying the distribution of 3981 differently expressed genes (DEGs) in GFLV-F13 (F13)- and GFLV-B844 (B844)-infected *Vitis vinifera* cv. Gewurztraminer vines compared to control plants. Number of up- and down-deregulated genes is represented in red and blue, respectively. Only one gene (in black) is regulated in the opposite direction. (**b**) Heatmap of the DEGs shown in (**a**). The color code indicates the fold change values in Log2 scale (Log2FC). (**c**) Distribution of genes specifically deregulated in Gw grapevines infected with strain B844 of GFLV. Number of induced (red) and repressed (blue) grapevine genes in the eleven top GO categories are shown. (Elim KS test, *p* ≤ 0.05).

To better understand the processes underlying the stunting phenotype of vines infected with GFLV strain B844, we analyzed the differentially expressed genes (DEGs) in the GFLV-F13- and GFLV-B844-infected vines compared to the controls. From the 25,255 genes identified, low counts, representing 4407 genes, were filtered out. A total of 3981 genes were differentially expressed (FDR ≤ 0.05), with 1458 genes (4.6% of the annotated PN40024 12X.v2 genes) deregulated by both virus strains (Figure 5a). This set of genes corresponded to the core genes reprogramed during GFLV infection. Noteworthy, from the 1458 shared genes, a single gene (VIT_208s0007g01690, predicted as a probable sugar phosphate translocator) was deregulated in the opposite direction, upregulated in the F13- and downregulated in B844-infected Gw. Thus, the phenotypic differences between the two strains are mainly associated with differentially deregulated genes rather than to the direction of the regulation of common genes.

A total of 2481 and 2958 genes were deregulated by GFLV-B844 and GFLV-F13, respectively. Whereas up- and downregulated genes were almost in even proportion following GFLV-F13 infection (48.2% upregulated vs 51.8% downregulated), a greater bias was observed towards downregulated genes following GFLV-B844 infection (40.4% upregulated vs 59.6% downregulated). Most of the significant changes (around 77%) are contained in a −1 and +1 Log2FC range (Figure 5b).

- Pathways differently affected by the two GFLV strains.

To identify pathways differently affected by the two GFLV strains, we further analyzed the DEGs with a special focus on the 1500 and 1023 genes specifically deregulated in the F13 and B844 treatments compared to the controls, respectively (Figure 5a). In order to decipher the affected biological processes, we performed a Gene Ontology (GO) terms enrichment analysis (Figure S3). The core DEGs shared by the F13 and B844 conditions (Figure S3a) belong to general-biological-process GOs, such as translation (ribosome biogenesis), growth (cell proliferation, regulation of meristem growth), and sugar metabolism (maltose metabolic process, starch biosynthetic process).

Globally, B844 DEGs (Figure S3d) focused on expression (positive regulation of transcription, DNA and RNA processing, translation), metabolism (carotenoid biosynthetic process), and defense pathways (response to chitin, regulation of plant-type hypersensitive response, RNA processing). The DEGs of the F13 treatment (Figure S3b) belong to expression pathways (DNA replication, RNA methylation) and the cell cycle and growth processes.

Remarkably, B844-specific DEGs (Figure 5c) participate in defense pathways, such as the regulation of HR, which is known to occur in plants in response to virus infection alongside specific symptom development.

F13-specific DEGs (Figure S3c) direct reprogramming to focus on replication, cellular development, and growth, and could be directly linked to the virus hijacking the cellular machinery for its multiplication.

These first results point to an HR-like response specifically occurring in Gw upon GFLV-B844 infection.

- Validation of the RNA-Seq differential genes expression by RT-qPCR.

To validate the RNA-Seq results, we randomly selected 20 genes (*AFC1*, *APL1*, *BCP*, *CDT1A*, *GAPDH*, *GPAT6*, *GRP*, *KCS5*, *NA_420*, *NA_1090*, *NA_1410*, *NEMFI*, *NQO1*, *PDF1*, *PDF2*, *PP2A*, *PP7*, *RD22*, *TIP41*, and *SYP121*) with different expression levels and regulation profiles and analyzed their expression by RT-qPCR using control samples as calibrators (Figure S4). Seven of these genes (*NEMFI*, *GAPDH*, *PP2A*, *NQO1*, *TIP41*, *AFC1*, *PP7*) were tested as potential reference genes using geNorm and Normfinder algorithms [53,54] to calculate their stability across all samples. All seven genes were indeed stable, with *TIP41* and *NEMFI* being the most stable across our eight samples. As already described in other virus–host pathosystems [63,64,65], *GAPDH*, which is commonly used as reference, did not perform well in our experimental setup. The RT-qPCR results of the remaining 19 genes were then analyzed using *NEMFI* as the reference gene. The overall expression profiles obtained by RT-qPCR were consistent with the RNA-Seq data (Figure S4), although the degree of modulation differed between the two techniques (Figure S4a–p). The results were contradictory only for three genes (*APL1*, *GAPDH*, and *NA_1090*, Figure S4q–s), possibly because of the higher variability of the RT-qPCR results for the F13 compared with the B844 treatment for these genes.

### 3.3. Biological Validation of an HR-Like Pathway Activation by GFLV-B844 in Gw Vines

#### Expression Analysis of Candidate Genes in Relation to PLANT Defense Pathways

In order to test the differential deregulation of the plant-type hypersensitive response by the two strains of GFLV, we extended our study to samples from the same field trial (Trial 1, Table S2), collected at two different time points, and samples from a second trial, established in a greenhouse (Trial 2). The symptoms developing on the plants from Trial 2 were consistent with our previous observations, with B844- and F13-infected vines showing stunting and mild symptoms, respectively. In total, 54 samples (36 from the field trial, including those analyzed by RNA-Seq, and 18 from the greenhouse) were subjected to RT-qPCR assays. Candidate genes (*NDF6*, *AGAL2*, *NF-YC4*, *AIL6*, *ERF4*, *SYP121*, and *CYP94*) were selected for their specific deregulation by GFLV-B844, with the highest induction or repression rates and/or their belonging to the GO “Regulation of hypersensitive response” (*ERF4*, *SYP121*, and *CYP94*; Supplemental Table S9). We also chose *PIN5* and *ROXY2*, which are deregulated by the two virus strains, but more substantially by B844 than F13. Finally, we chose *PR10* and *RDR1*, two defense genes that showed a low induction by both viruses in our transcriptomic analysis.

**Figure 6:**
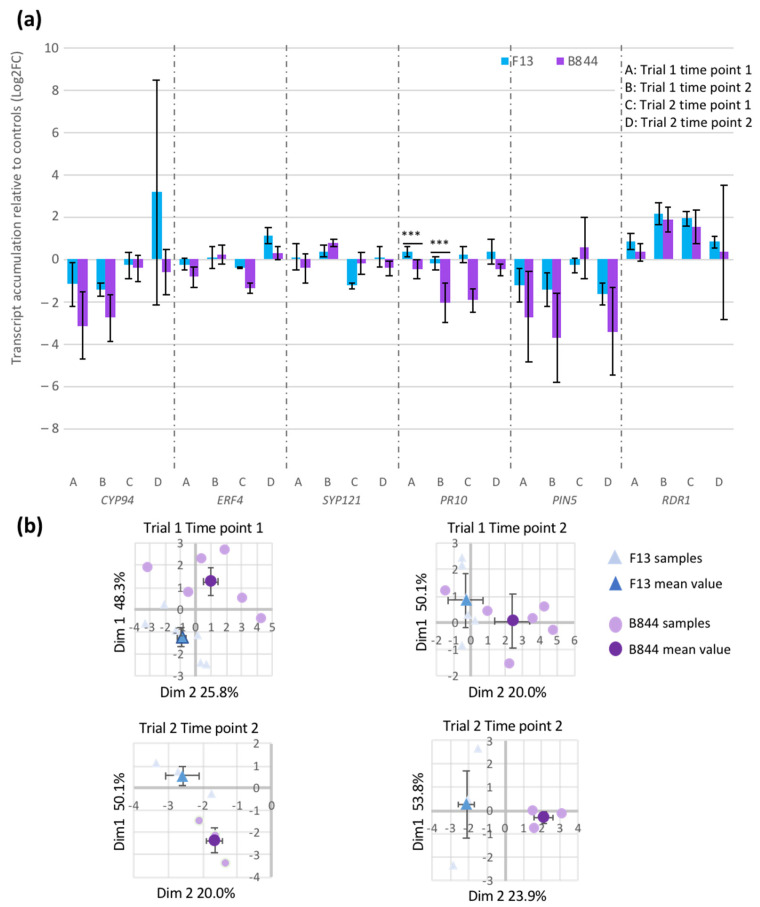
Comparative transcript accumulation of candidate genes in grapevine fanleaf virus (GFLV) strains B844 (B844) and F13 (F13)-infected *Vitis vinifera* cv. Gewurztraminer (Gw) vines grown in different conditions and collected at different pheno¬logical stages. (**a**) Of the eleven selected genes analyzed by RT-qPCR, six genes (*CYP94, ERF4, SUP121, PR10, PIN5*, and *RDR1*) are shown. These six genes are involved in the plant defense or regulation of HR. Histograms represent mean values of Log2 fold changes compared to control plants. *n* = 6 for Trial 1, and *n* = 3 for Trial 2 (see Table S2 for a description of the samples). Error bars = standard deviation. Statistically significant differences (Mann–Whitney rank test) are indicated with asterisks: *** *p* < 0.005. (**b**) Principal component analysis (PCA) of the expression level of the eleven tested genes. Loading scores on the PC1 and PC2 axis are shown in the percent of variability in the original data. Analysis was performed using software package Statgraphics Centurion 15.1.02 (Stat Point technologies Inc., Warrenton, VA, USA).

Significant variations in the expression of all eleven genes selected for this study were apparent in relation to the environmental conditions and the phenological stages, suggesting highly dynamic processes in gene regulation (Figure 6 and Figure S5). However, some differences were virus-strain-specific, as shown by the RT-qPCR results analyzed either by gene (Figure 6a), or globally by the PCA of the eleven genes that enables the discrimination of the virus strains, regardless of the trial and time point considered (Figure 6b). Notably, the expression of PR10 (Figure 6a) appeared differently regulated by strains B844 and F13. Altogether, these results illustrate that some genes involved in the HR, or its regulation, are differently expressed in plants infected with GFLV-B844 compared with plants infected with GFLV-F13.

- Metabolomic analyses.

**Figure 7:**
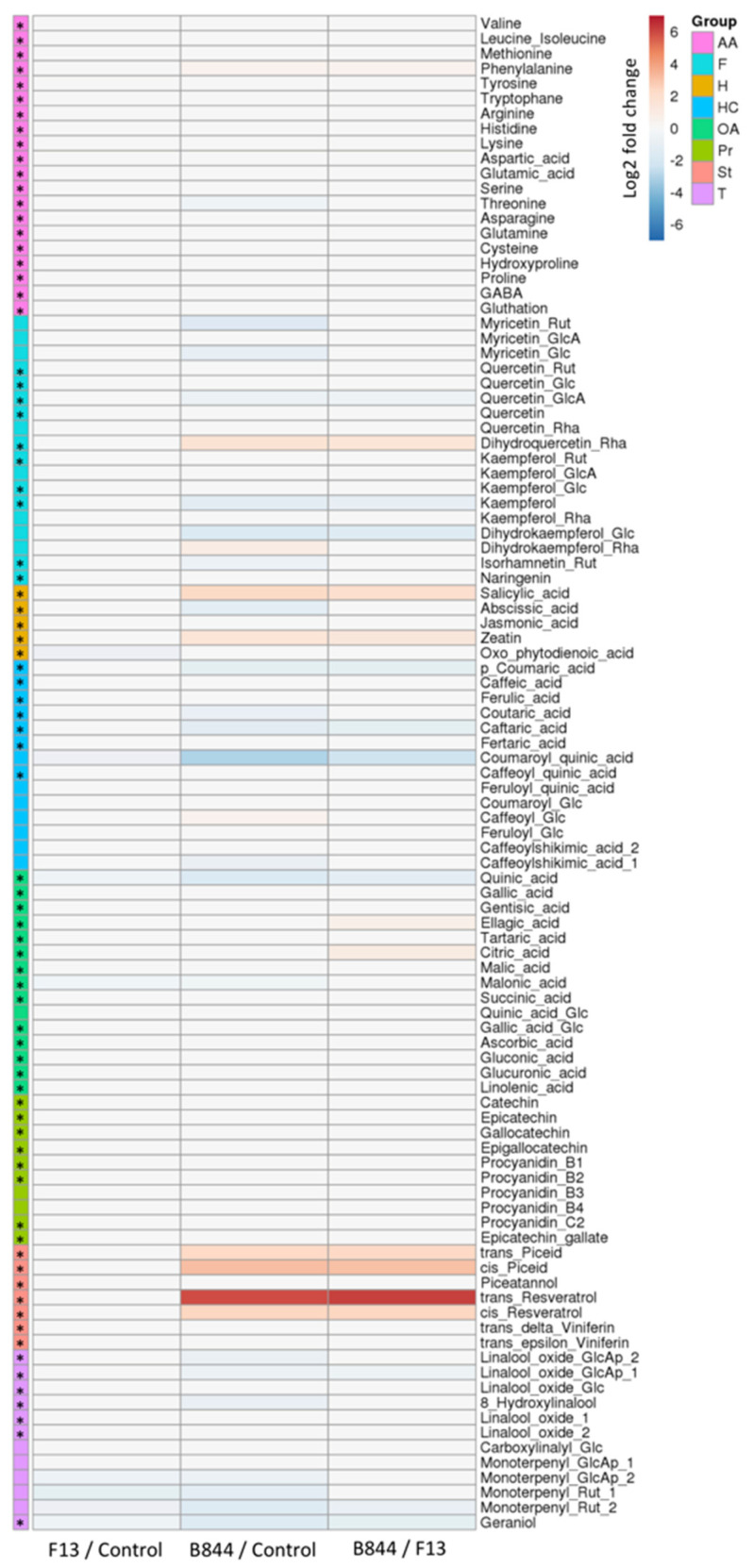
Impact of grapevine fanleaf virus (GFLV) strains F13 and B844 on leaf metabolites. Pairwise comparison of significant metabolite changes in leaves of *Vitis vinifera* cv. Gewurztraminer (Gw) infected with the two different GFLV strains, and in the control Gw leaves. Log2 of significant metabolite fold changes for indicated pairwise comparisons are given by shades of red or blue colors, according to the scale bar. Metabolites were grouped according to their chemical or functional family as amino acids and derivatives (AA), flavonoids (F), hormones (H), hydroxycinnamic acids and derivatives (HC), organic acids (OA), proanthocyanidins (Pr), stilbenoids (St), and terpenoids (T). Data represent mean values of 35–38 biological replicates for each condition (See Table S2 for a precise description of the samples). Statistical analysis was performed using Tukey’s honest significant difference method, followed by a false discovery rate (FDR) correction, with FDR < 0.05. For FDR ≥ 0.05, Log2 fold changes were set to 0. Glycosides are indicated as glucoside (Glc), apiosylglucoside (GlcAp), glucuronide (GlcA), rhamnoside (Rha), or rutinoside (Rut). * indicates that metabolite identity was confirmed with the corresponding authentic standard.

To gain further insights into the grapevine response to GFLV infection, a metabolomic analysis was performed by UHPLC-MS on leaf samples collected at different time points from all three treatments grown in three different trials (Table S2). In total, 112 samples from 36 plants were subjected to a targeted metabolomic analysis in order to quantify the relative amounts of 101 compounds belonging to major chemical or functional families, including amino acids and derivatives, flavonoids, hormones, hydroxycinnamic acids and derivatives, organic acids, proanthocyanidins, stilbenoids and terpenoids. A pairwise comparison of the significant metabolite changes in the control leaves and leaves from the GFLV-infected vines revealed major differences in the plant responses to the B844 or F13 strains (Figure 7 and Figure S6). The leaves of the control and the GFLV-F13-infected vines exhibited very similar profiles, indicating that infection by this mild GFLV strain had very little impact on the selected leaf metabolites. Similarly, infection with GFLV-B844 had no significant effect on the majority of the selected metabolites. However, GFLV-B844 impacted a small number of metabolites belonging to specific families, when compared with both the control and GFLV-F13-infected plants. GFLV-B844 had a moderate impact on some metabolites belonging to flavonoids and hydroxycinnamic acids. Interestingly, both salicylic acid (SA) and zeatin were significantly increased by factors of 5 and 2.5, respectively, in GFLV-B844-inoculated vines, whereas jasmonic acid (JA) remained unaffected. Nevertheless, the most remarkable impact of GFLV-B844 infection was a very significant accumulation of phytoalexins, such as piceid and resveratrol, with a sixty-fold increase in the *trans*-resveratrol amount compared with the control or GFLV-F13-infected plants.

The application of the cytokine zeatin has been shown to decrease symptoms caused by the bacteria, *Pseudomonas syringae*, in tobacco plants, in relation to increased levels of SA accumulation [66]. Resveratrol and its glycosylated derivatives, *trans*-piceids, have been shown to have antimicrobial properties, among others [67], and resveratrol has been more precisely associated to the hypersensitive response in *Vitis* cells [68,69,70].

Altogether, these results suggest that GFLV-B844, the severe strain, stimulates the chronic accumulation of specific metabolites related to the plant defense pathways in a way typical of a biotrophic pathogen.

- Virus restriction

**Figure 8:**
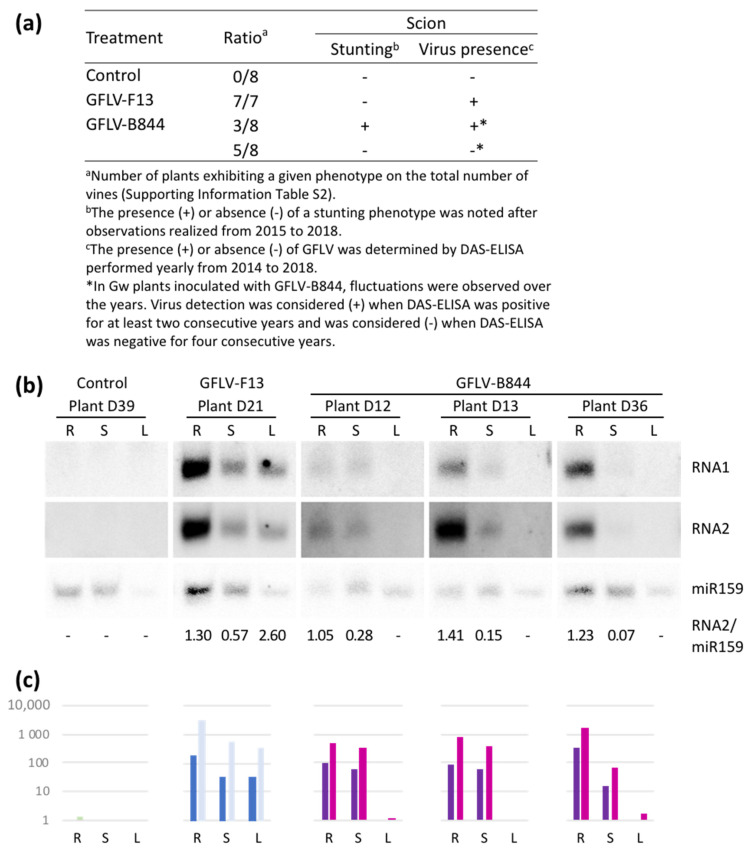
Restriction of grapevine fanleaf virus (GFLV) strain B844 in some *Vitis vinifera* cv. Gewurztraminer (Gw)-grafted vines. (**a**) Virus accumulation and symptom development. (**b**) Northern blot detection of small viral RNAs in the rootstock (R) just below the graft union, scion (S) just above the graft union, and leaves of the scion (L) in five plants. Probes detect small RNAs deriving from RNA1 and RNA2 of GFLV and the miR159 as a loading control. Ratios of RNA2 over miR159 signal intensities, as determined with ImageJ, are given below the respective lanes. (**c**) Number of GFLV-RNA1 (dark colors) and -RNA2 (light colors) molecules per ng of total RNA (×1000 mol/ng), as assessed by RT-qPCR analysis in the same samples, as in (**b**).

HR is mostly associated with a restriction of the pathogen’s systemic spread within the plant, although the induction of HR has been described to be insufficiently effective at limiting the propagation of some viruses [35,71]. In another field trial, established in 2013 (Trial 4, Table S2), some B844-inoculated vines showed an unusual phenotype (Figure 8a): three plants exhibited a classical stunting phenotype and tested positive for GFLV in the DAS-ELISA, although with some year-to-year fluctuations, while the remaining five plants had no obvious phenotype and tested negative in the DAS-ELISA. F13-inoculated vines showed mild symptoms and consistently tested positive in the DAS-ELISA, as expected.

To check for the presence of the virus, three randomly chosen symptomless plants were further analyzed by RT-qPCR and northern blot using RNA extracted from cortical scrapings, after removing the bark of: (i) the rootstock below the graft union; (ii) the scion immediately above the graft union; and (iii) the leaves of the scion. The presence of viral RNAs and their catabolic products (viral small RNAs, vsiRNAs) was investigated by RT-qPCR and northern blot, respectively (Figure 8b,c). VsiRNAs originating from both RNA1 and RNA2 were detected in the rootstock and in the scion immediately upstream of the graft union point but were absent from the apical leaves (Figure 8b). These results were confirmed by RT-qPCR analyses (Figure 8c), showing that the severe strain, GFLV-B844, was present in all rootstocks but was unable to translocate to the upper parts of the scion of some plants. In these plants, the virus was efficiently restricted to the rootstock and the plants remained symptomless, showing a high degree of resistance toward the severe strain.

## 4. Discussion

GFLV causes variable symptoms in the vineyard, depending on the virus strain, grapevine genotype, cultural practices, and environmental conditions [28]. A study of the virus symptomatology on this perennial woody fruit crop in the vineyard is complicated by the frequency of multiple virus infections. In this work, we describe a unique experimental setting where two grapevine genotypes were graft-inoculated with five distinct GFLV strains and monitored during three to five growing seasons. This setting allowed us to distinguish the GFLV strain, B844, which caused a drastic yield reduction and stunted growth in Gw grapevines from other strains. From these observations, GFLV-B844 is considered hyper-aggressive on Gw, whereas F13 is a milder strain, thus partially confirming previous results (Legin et al., 1993). The virus titer did not consistently and significantly vary with the viral strain and phenotype, showing that the virus concentration cannot explain the severity of the GFLV-B844-induced symptoms.

We compared the transcriptomic changes induced by the severe strain, GFLV-B844, and the mild strain, F13, using HTS because (i) it is an unbiased approach to quantifying gene expression, and (ii) it can ascertain the infection status of grapevines. Apart from some GRSPaV, GYSVd, and HSVd reads, we did not retrieve any unexpected sequences from virus or viroid origin, validating our conditions to produce vines with single-GFLV-strain infections, and controlled settings that prevented a superinfection of the experimental vines maintained in the open and in the greenhouse. Thus, although we cannot completely rule out a synergistic effect of GRSPaV, GYSVd, or HSVd on GFLV symptoms, it is very likely that the GFLV-B844 is responsible for the more severe symptoms on Gw vines. This represents the first example of a study on virus-induced symptoms in grapevine with a nontargeted search for viral sequences. By comparing the transcriptomic changes caused by the hyper-aggressive strain, B844, with those caused by the milder strain, F13, we identified 2481 deregulated unigenes, 1458 of which were shared by the two strains, and 1023 that were specifically modulated by strain B844 (419 induced and 604 repressed genes). The RNA-Seq results were confirmed by RT-qPCR on 16 genes out of the 19 randomly chosen gene candidate targets. Although an outlier plant was detected and removed from further analyses, no DEG was lost.

Among the GOs of deregulated genes found in this work, some are often found in the transcriptomic profiling of viral infections. This is the case for genes involved in photosynthetic processes, ribosome biogenesis, sugar metabolisms, and translation [72,73,74]. The perturbation of photosynthesis and the chloroplasts has already been described for viral infections in general [75,76], and GFLV in particular [77,78]. The stunting symptoms of strain B844 could be consistent with the repressed genes implicated in the regulation of meristem growth. The two top GOs of the 43 B844-DEGs (namely, the response to chitin and the regulation of the plant-type hypersensitive response) are related to plant defense and, more precisely, to the hypersensitive-type response. This was of particular interest because it led to the attractive hypothesis that HR might have taken place in GFLV-B844-infected ‘Gewurztraminer’ vines. This hypothesis is reinforced by the specific accumulation of the major vine phytoalexin, resveratrol, and the signaling hormone, SA, in GFLV-B844-inoculated Gw vines, and by the limited virus spread in some plants. Phytoalexins are low molecular mass secondary metabolites with large spectrum antimicrobial activities. Resveratrol, the main phytoalexin in the *Vitis* spp., has been associated with hypersensitive or hypersensitive-like responses in *Vitis* cells upon fungus elicitation [68,69,70]. An antiviral effect of resveratrol or its derivatives has been mentioned for human cells infected with the now-called SARS-CoV-1 [79] and, very recently, against tobacco mosaic virus infection in herbaceous plants [80]. Whether resveratrol or trans-resveratrol has a direct antiviral effect and contributes to the success of HR against GFLV remains to be tested.

HR occurs when the product of a cellular resistance gene (R) interacts with a pathogenic avirulence factor (Avr). This interaction generally leads to cell death and a limitation of the virus movement within the plant [81,82,83,84]. However, cell death and virus restriction represent separate processes that have been temporally, spatially, or genetically uncoupled for a few plant–virus pathosystems [71,85,86,87,88,89]. The efficiency of resistance was suggested to be correlated with the speed and the intensity of the host response. A rapid reaction of the plant would thus promote an efficient restriction of the virus without severe necrosis, whereas a slower reaction would be unable to stop virus movement, and this may increase the intensity of the reaction and result in systemic HR. Cell death could, therefore, be regarded as a side effect of a delayed defense response [71]. No necrosis was observed in our pathosystem. However, the virus entered the Gw genotype through the vascular system via the graft-inoculation procedure used in this study. Thus, we do not know whether necrosis took place in some plants or not.

A study of the specific role of deregulated genes could now give a better picture of the reactions leading to the plant’s response and the observed phenotype. This specific involvement is difficult to predict from the literature because (i) our pathosystem results in a long-lasting contact between the virus and its host and it is, therefore, difficult to evaluate an induction step from a feed-back regulation of the induced reaction, and (ii) some genes have been described with the opposite function in different pathosystems. This is well-exemplified by the transcription factor, WRKY 11, found to be decreased in the GFLV B844-infected grapevines of the present work. It has recently been established as a positive regulator of resistance to *Botrytis cinerea* in strawberry fruit [90], and is known like WRKY7, as a negative regulator of resistance to *P. synringae* in *Arabidopsis thaliana* [91,92]. Thus, although some of the DEGs have described functions in *A. thaliana* regarding stress responses, it is very speculative to predict their role in our pathosystem.

Timing and R-Avr interaction (affinity or quantity) are predicted to determine the outcome of the plant–virus interaction in a continuum of possible responses, ranging from extreme resistance to systemic HR [71]. Hence, HR and its downstream regulation are not contradicted by the systemic infection observed in many of the plants of our experiment. The grafting procedure represents a high-pressure virus inoculation method [18] that could influence the onset and efficiency of the defense response. Moreover, we might have introduced a strong bias against the proportion of plants actually restricting the virus because we generally select plants for the virus presence after grafting and before greenhouse or field establishment. An HR response to GFLV, only capable of efficiently restricting the virus in a fraction of the inoculated plants, has been shown to occur in *Nicotiana occidentalis* when inoculated with strain F13. It is not known what differs between *N. occidentalis* plants, where resistance occurs, and plants showing a so-called trailing HR, where the pathogen is not completely stopped and initiates a reaction in all the tissues where it moves. In this herbaceous model plant, the molecular markers of the HR were detected starting three days post inoculation [35]. Because the inoculation procedure of grapevines requires the in vitro heterologous grafting of a rootstock onto an infected *C. quinoa* stem, followed by the herbaceous grafting of a grapevine cultivar onto the infected rootstock, the experiments lasted several years after the inoculation. This may explain why our transcriptomic analysis did not show an induction of the characteristic HR genes, for instance, the homologs of HSR203J, NDR1, or EDS1, as these genes are expressed at early time points after pathogen challenge [93]. To confirm that GFLV-B844 induces an HR in *V. vinifera* cv. Gewurztraminer, a model of this pathosystem, allowing a direct inoculation of the virus, needs to be developed.

Stunting, as a consequence of plant defense in perennial or woody plants, where mechanistic data is scarce, has only begun to be mentioned in the literature. A recent paper describes the upregulation of genes implicated in the biosynthesis of phytoalexins in response to apple replant disease [94]. Another example is a stunting phenotype that has been described in hop plants infected with a viroid [95]. These plants show a deregulation of HR-related genes. Here, by comparing plants infected with a mild and a severe GFLV strain, we provide an additional indication that HR could be associated with severe stunting symptoms. A comparison of the plants exhibiting effective virus resistance with the plants presenting an ineffective defense response can now be envisioned in both the herbaceous model plant, *N. occidentalis,* and the woody crop grapevine. This should help understand what component of the HR response is actually responsible for the virus resistance.

## 5. Conclusions

To conclude, our work used a unique experimental setting of mono-infected vines for the identification of GFLV strains with distinct symptoms on Gewurztraminer. This has provided the tools to compare the transcriptome of plants infected by a severe (B844), and a mild strain (F13), of GFLV in order to shed light on the mechanisms underlying pathogenesis. From the specifically deregulated genes identified in our study, we hypothesize that an HR-type reaction might be induced by the severe strain causing stunting. The accumulation of SA and phytoalexins, and the partial resistance observed in some GFLV-B844 infected grapevines, strengthen our hypothesis. This defense reaction might lead to either a severe stunting or to resistance, most probably by the action of SA, which is involved in the defense towards viral diseases, as well as in dwarfing symptoms during virus infection in herbaceous plants [75]. Thus, SA likely represents the major player in the growth/immunity tradeoff in grapevine, in a manner comparable to what has been described in Arabidopsis [96,97]. To the best of our knowledge, this is the first indication of HR in virus-infected grapevines and the potential mechanisms underlying stunting in a perennial woody fruit crop.

## Figures and Tables

**Table 1:** Summary of Illumina sequencing and read mapping to the grapevine genome.

Treatment ^a^	Plant ^b^	Total Reads ^c^	Mapped Reads ^d^	% ^e^	Unique Reads ^f^	% ^e^	Reads Assigned to a Gene ^g^	% ^e^
Control	B31	51,964,098	42,741,560	82	42,153,543	81	40,248,531	77
	B34	59,910,092	52,451,408	88	51,647,516	86	49,130,979	82
	C51	65,724,896	56,105,085	85	55,354,647	84	52,784,724	80
F13	B52	69,714,014	59,901,468	86	58,981,711	85	56,246,251	81
	B53	75,771,722	66,064,642	87	65,065,605	86	62,069,793	82
	C38	85,633,030	74,306,333	87	73,166,998	85	70,037,177	82
B844	B47	165,443,500	139,722,683	84	137,021,434	83	129,811,616	78
	C39	54,436,852	46,539,167	85	45,791,912	84	43,348,284	80
	C40	58,344,878	49,214,165	84	48,446,050	83	46,189,830	79

^a^ Gw grapevine leaves of non-inoculated (control, in green color), GFLV-F13-inoculated (F13, in light blue color), or GFLV-B844-inoculated plants (B844, in purple color). ^b^ Individual plants are considered biological replicates. ^c^ Total number of reads. ^d^ Number of reads mapping to the grapevine PN40024 (12X.v2) reference genome. ^e^ Percentage of total mapped reads over total reads. ^f^ Number of reads mapping to a unique position on the PN40024 12X.v2 reference genome. ^g^ Number of reads assigned to PN40024 (12X.v2) gene annotation.

## Data Availability

RNA-Seq data is being deposited in GEO (under progress). Other data that does not appear in the Supplementary Materials is available upon request.

## Supplementary Materials

The following are available online at https://www.mdpi.com/article/10.3390/v13112138/s1: Figure S1: Annual comparison of grapevines infected with GFLV strains F13 and B844; Figure S2: Phenotype of the plants subjected to RNA-Seq analysis; Figure S3: GO categorization of differently regulated genes; Figure S4: Comparison of RNA-Seq and qRT-PCR determination of transcript accumulation; Figure S5: Transcript accumulation of five candidate genes, not related to defense, in infected vines grown in different conditions and collected at different time points; Figure S6: Principal component analysis of the global impact of GFLV strains F13 and B844 on leaf metabolites of GFLV-infected vines; Table S1: GenBank accessions of grapevine fanleaf virus strains used in this study; Table S2: Trials and plant analyzed in this study; Table S3: Primers used in RT-qPCRs performed on cellular transcripts; Table S4: Annual symptom scores of the five GFLV strains on the two grapevine cultivars; Table S5: Annual and cumulative fruit yield reduction caused by the five GFLV strains on the two grapevine cultivars; Table S6: Effect of strains F13 and B844 on the growth of ‘Gewurztraminer’ and ‘Chardonnay’ grapevines; Table S7: Read counts and RPKM of viral and viroidal sequences obtained in the RNA-Seq experiment; Table S8: Details on excluded reads; Table S9: Grapevine genes specifically deregulated in response to GFLV-B844 infection of ‘Gewurztraminer’ vines.

## Abbreviations

AFC1	Serine/threonine-protein kinase afc1
AGAL2	Alpha galactosidase
AIL6	Ap2-like ethylene-responsive transcription factor ail6
APL1	ADP-glucose pyrophosphorylase large subunit
BCP	Blue copper protein
CDT1A	cdt1-like protein chloroplastic-like
Ch	Chardonnay
CYP94	cytochrome p450 CYP94B3
ERF4	Ethylene Response Factor 4
GAPDH	Glyceraldehyde-3-phosphate dehydrogenase
GFLV	Grapevine fanleaf virus
GPTA6	Glycerol-3-phosphate acyltransferase 6
GRP	Glycine rich protein
GRSPaV	Grapevine rupestris stem pitting-associated virus
Gw	Gewurztraminer
GYSVd	Grapevine yellow speckle viroid
HR	Hypersensitive reaction
HSVd	Hop stunt viroid
HTS	High throughput sequencing
KCS5	3-ketoacyl- synthase
NA	Not annotated
NDF6	NAD(P)H dehydrogenase complex
NEMF1	Nuclear export mediator factor nemf-like
NFYC4	Nuclear transcription factor y subunit c-4
NQO1	Quinone oxidoreductase 1
PDF1	Protodermal factor
PDF2	Defensin-like protein 6-like
PIN5	Auxin efflux carrier component
PP2A	Serine/threonine protein phosphatase 2A (PP2A) 65 KDa regulatory subunit A
PP7	Serine/threonine-protein phosphatase 7
PR10	Pathogenesis related protein 10
RD22	Dehydration-responsive protein rd22
RDR1	RNA polymerase RNA dependante
ROXY2	Glutaredoxines ROXY2
SYP121	Syntaxin-related protein nt-syr1
TIP41	Tip41-like protein
VST	Variance-stabilized transformation
