# Supplementary material for: Severe Stunting Symptoms upon Nepovirus Infection Are Reminiscent of a Chronic Hypersensitive-like Response in a Perennial Woody Fruit Crop
Source: Viruses. 2021 Oct 22;13(11):2138. doi: 10.3390/v13112138 (PMC8625034; doi:10.3390/v13112138)
Supplement: Supplementary file 1 [file viruses-13-02138-s001.zip › viruses-1408418-Supplementary Figures conversion.pdf]

viruses

MDPI

Supplementary Materials

Severe Stunting Symptoms upon Nepovirus Infection Are Reminiscent of a Chronic Hypersensitive-Like Response in a Perennial Woody Fruit Crop

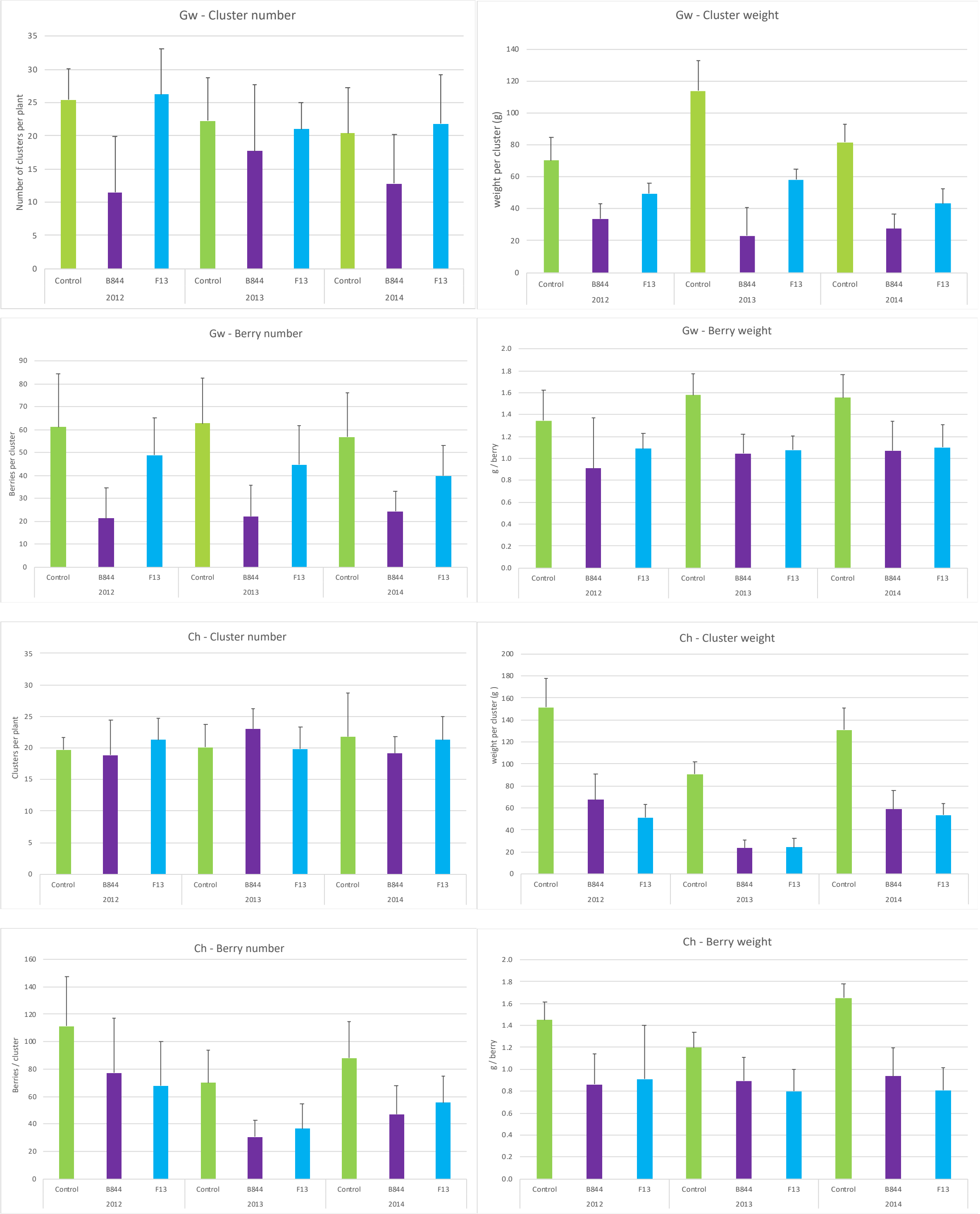

Figure S1. Annual comparison of grapevines infected with GFLV strains F13 and B844. Gw (**a to d**) and Ch (**e to h**) vines infected with GFLV strain F13 or B844 were monitored from 2012 to 2014 for cluster number **(a, e)** and weight **(b, f)**, for berry number **(c, g)** and weight **(d, h)**. Error bars = standard deviation. Statistically significant differences (Mann-Whitney-test) are indicated with asterisks (*: *p* <0.05, **: *p* <0.01 ****p* <0.005).

(a) **(b)**

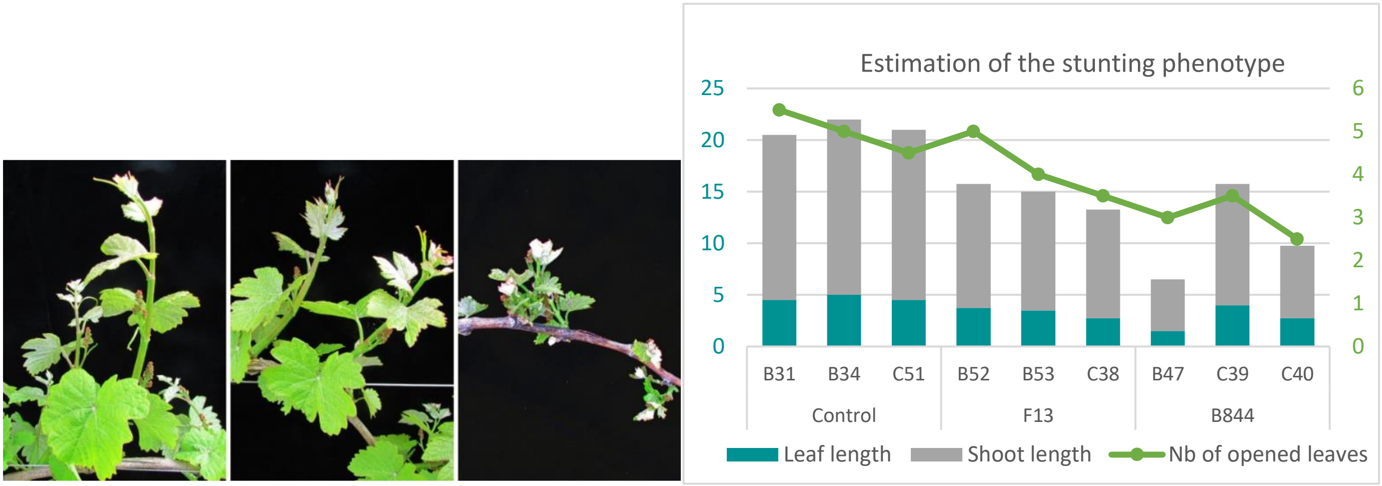

Figure S2. Phenotype of the plants subjected to RNA-Seq analysis. **(a)** Photographs showing leaves of representative non-inoculated (control, plant B33), GFLV-F13 inoculated (F13, plant B52) and GFLV-B844 inoculated (B844, plant B47) scions were taken at the same phenological stage. **(b)** The stunting phenotype of the plants used for transcriptomic analyses was assessed by counting the number of opened leaves and measuring the size of the leaves and shoots.

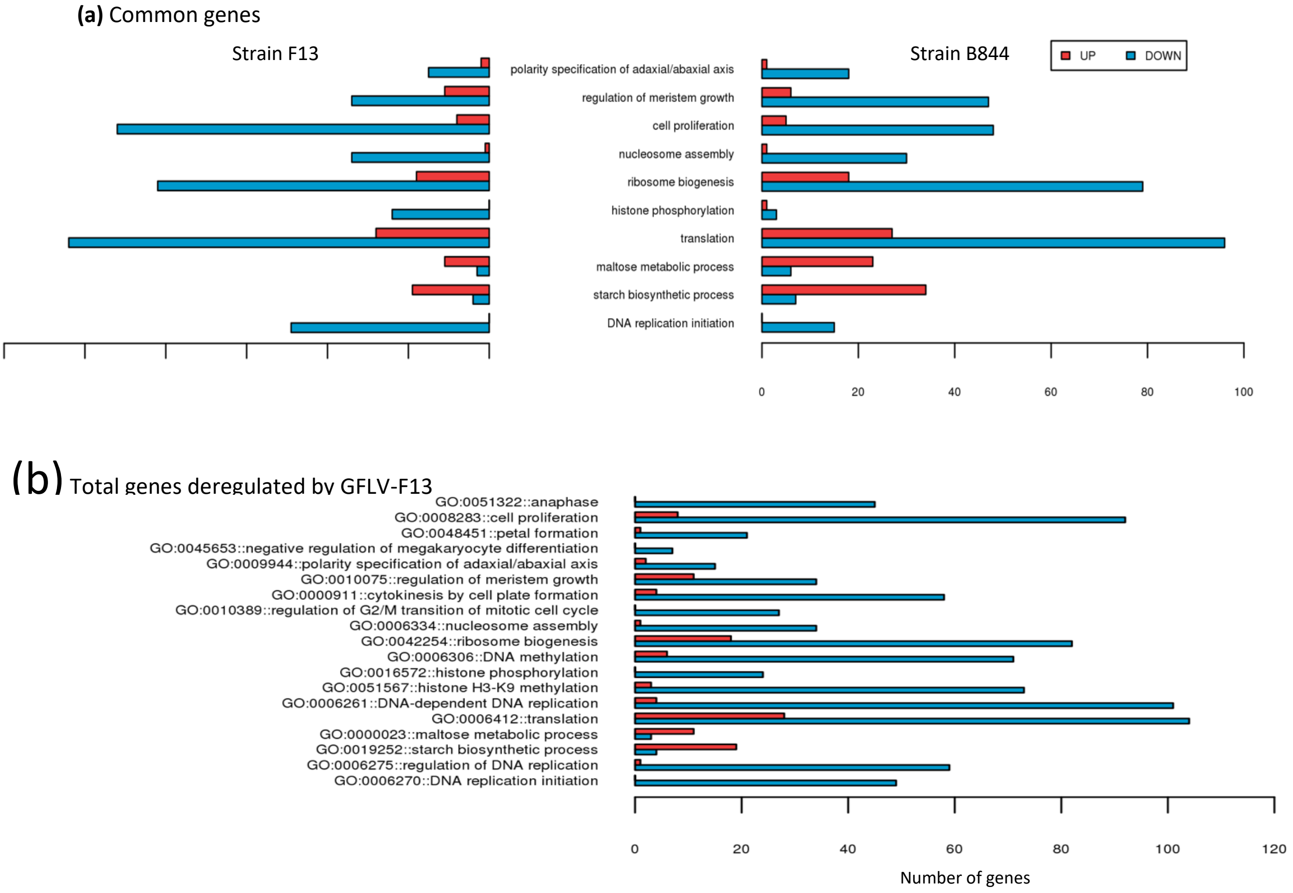

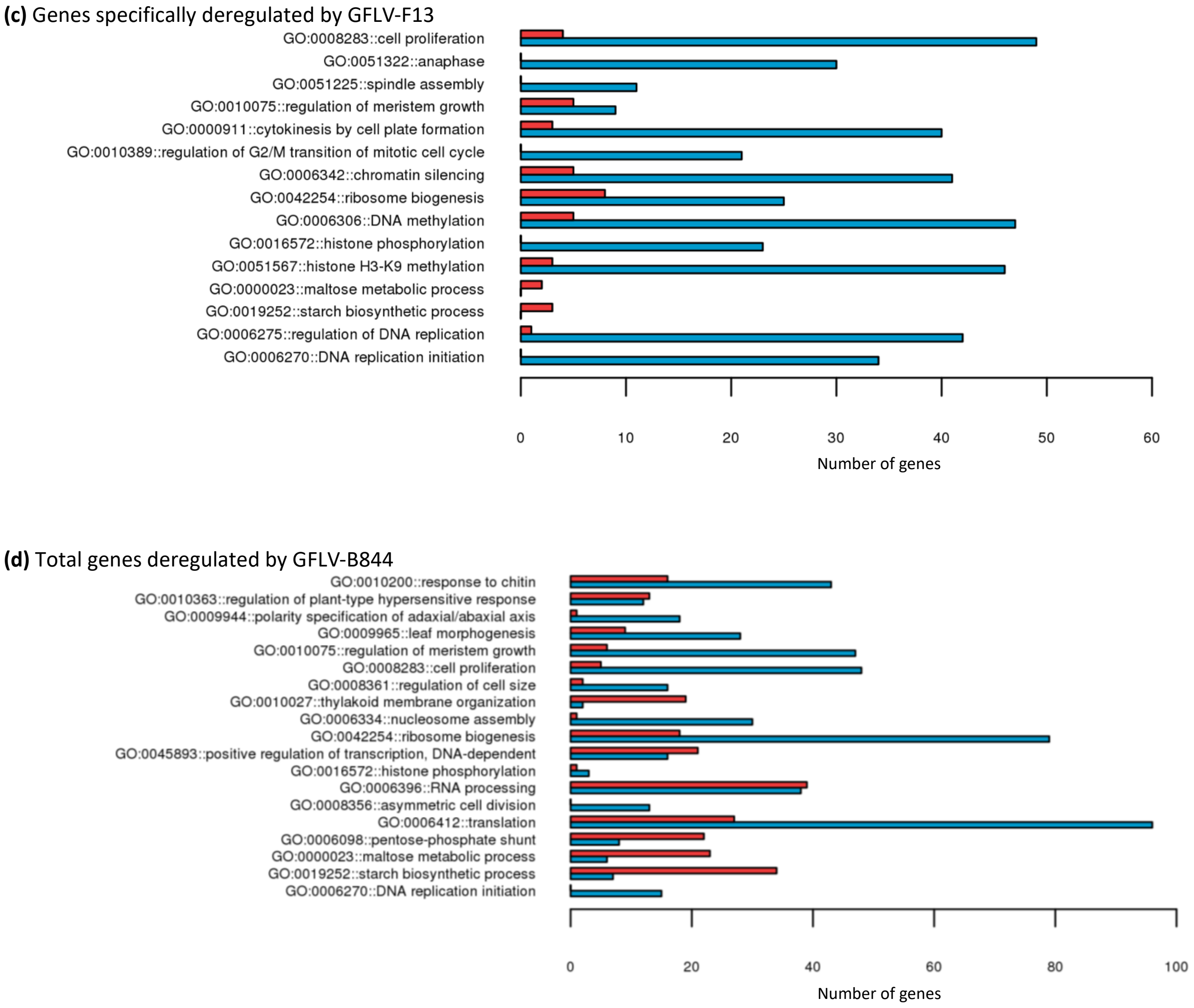

Figure S3. GO categorization of DEGs. GO term enrichment of the genes deregulated in response to both GFLV-B844 and -F13 **(a)**, to GFLV-F13 **(b, c)**, or to GFLV-B844 **(d)**. Numbers of induced (red) and repressed (blue) grapevine genes in the top biological process GO categories are shown. (Elim KS $P \leq 0.05$).

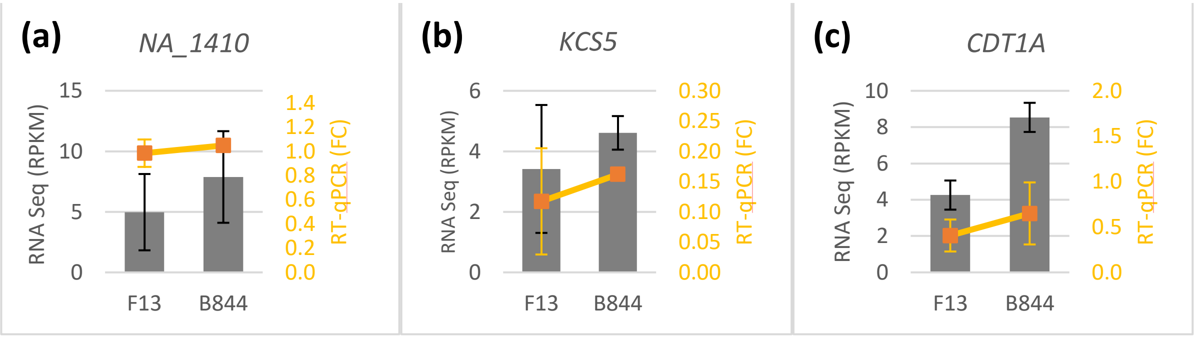

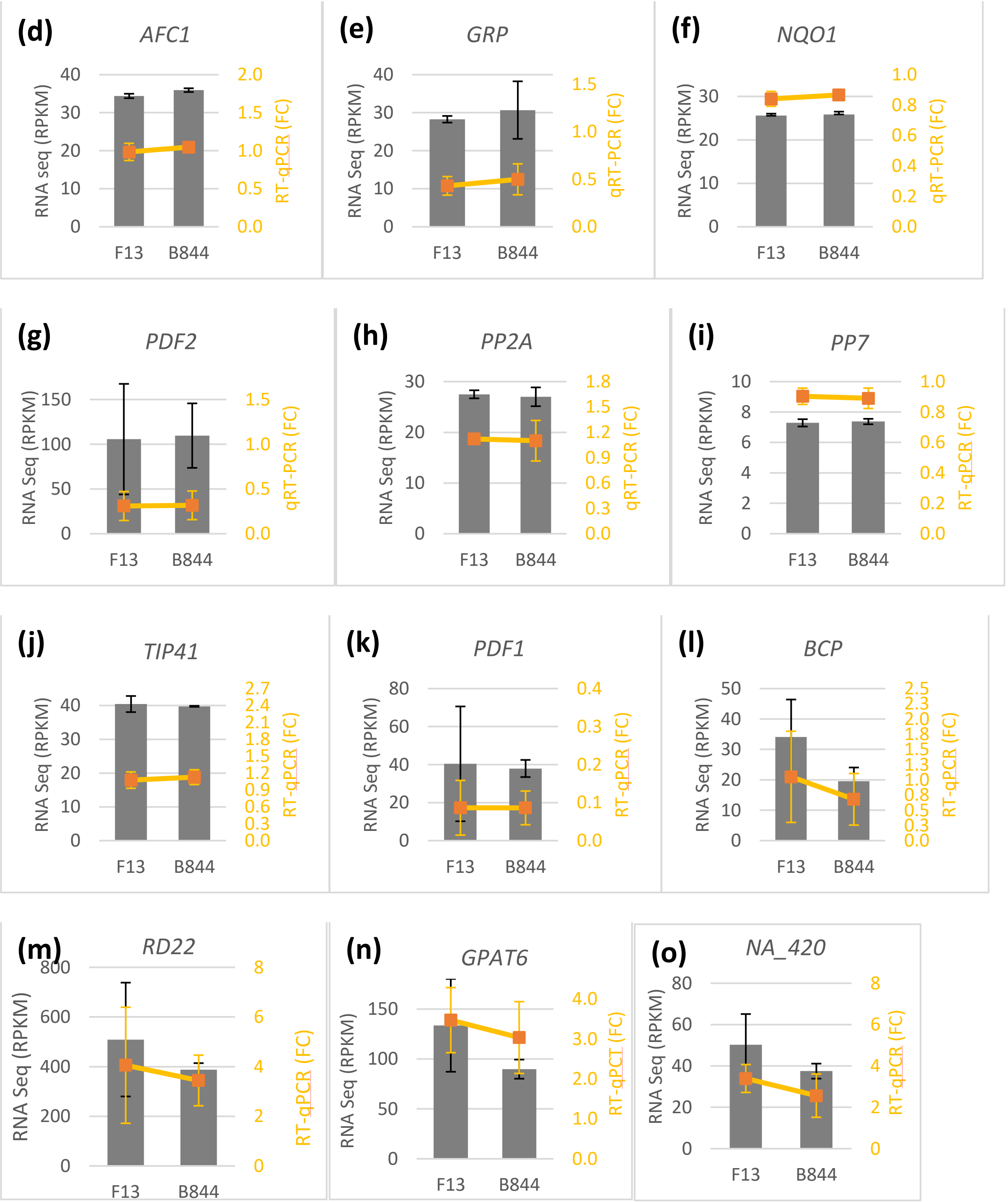
(d)
AFC1
RNA seq (RPKM)
RT-qPCR (FC)
F13
B844
(e)
GRP
RNA Seq (RPKM)
qRT-PCR (FC)
F13
B844
(f)
NQO1
RNA Seq (RPKM)
qRT-PCR (FC)
F13
B844
(g)
PDF2
RNA Seq (RPKM)
qRT-PCR (FC)
F13
B844
(h)
PP2A
RNA Seq (RPKM)
qRT-PCR (FC)
F13
B844
(i)
PP7
RNA Seq (RPKM)
RT-qPCR (FC)
F13
B844
(j)
TIP41
RNA Seq (RPKM)
RT-qPCR (FC)
F13
B844
(k)
PDF1
RNA Seq (RPKM)
RT-qPCR (FC)
F13
B844
(l)
BCP
RNA Seq (RPKM)
RT-qPCR (FC)
F13
B844
(m)
RD22
RNA Seq (RPKM)
RT-qPCR (FC)
F13
B844
(n)
GPAT6
RNA Seq (RPKM)
RT-qPCT (FC)
F13
B844
(o)
NA_420
RNA Seq (RPKM)
RT-qPCR (FC)
F13
B844

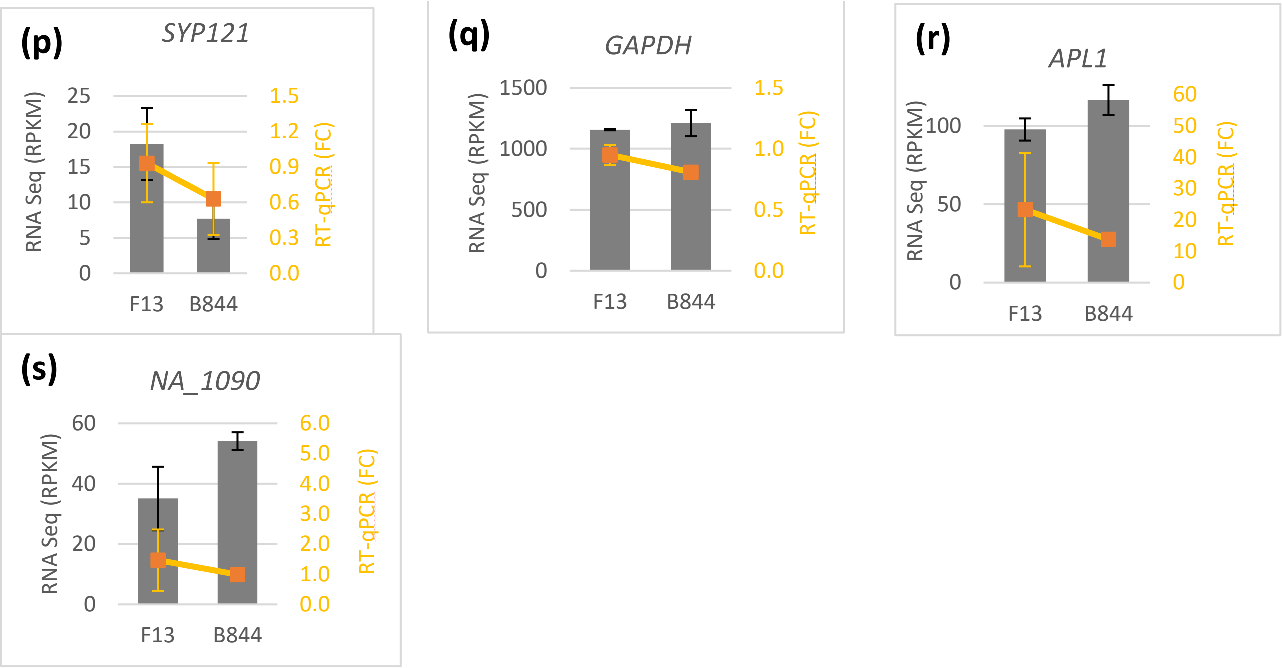

Figure S4. Comparison of RNA-Seq and qRT-PCR determination of transcript accumulation. Expression profiles of 19 randomly chosen *Vitis* genes determined by RNA-Seq (histograms) and RT-qPCR (lines) are represented as means and standard deviations of three (GFLV-F13) or two (GFLV-B844) biological replicates. Normalization for RT-qPCR used *NEMFI* as reference gene and control plants as calibrators.

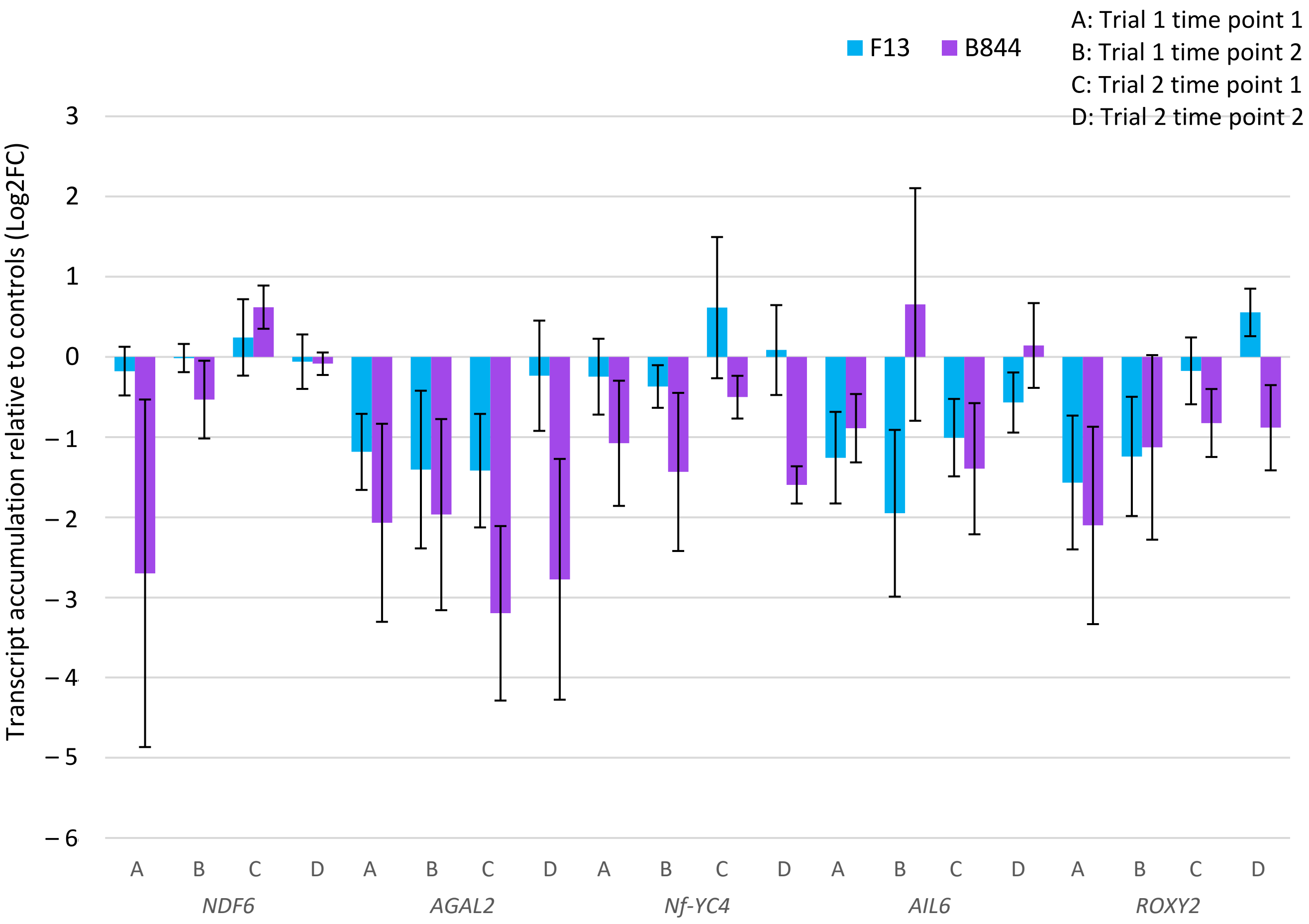

Figure S5. Transcript accumulation of candidate genes in GFLV-B844 vs -F13 infected Gw vines grown in different conditions and collected at different time points. Of the eleven genes analyzed by RT-qPCR, five genes not related to defense are shown here. Histograms show mean values. Error bars = standard deviation. Statistically significant differences (Mann-Whitney rank test) are indicated with asterisks: $**p < 0.01$.

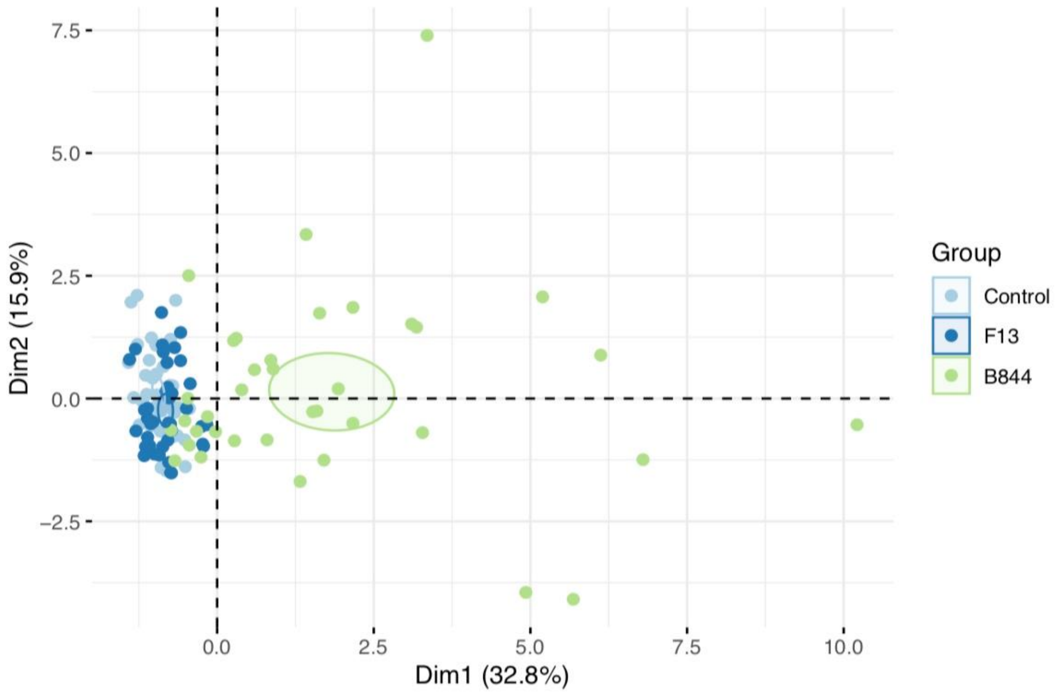

Figure S6. : Global impact of GFLV strains F13 and B844 on leaf metabolites. Principal component analysis performed on amounts of all analyzed compounds in uninoculated Gw controls and Gw infected with GFLV strains F13 or B844 . For each group, 35 to 38 biological replicates were used. The first two principal components explain 32.8 % and 15.9 % of the variance separating the different groups of plants, respectively.
